# Age and sex modify the association between secondhand smoke exposure and self-reported asthma in school-aged children (6–11 years): a cross-sectional study from NHANES 2005–2020

**DOI:** 10.1186/s12889-026-28663-9

**Published:** 2026-08-03

**Authors:** Shanshan Xiao, Tianxin Hu, Chunyan Du, Lijuan Bai, Ke Dong, Wentao Guo, Xun Jiang

**Affiliations:** 1 Department of Pediatrics, Tangdu Hospital, The Fourth Military Medical University, No.569 XinsiRoad, Baqiao District, Xi’an, Shaanxi 710038 China; 2 Department of Pharmacy, Tangdu Hospital, The Fourth Military Medical University, Xi’an, China; 3 Department of Clinical Laboratory, Tangdu Hospital, The Fourth Military Medical University, Xi’an, China

**Keywords:** Secondhand smoke exposure, Childhood asthma, Effect modification, School-aged children, NHANES

## Abstract

**Background:**

Secondhand smoke (SHS) is a known risk factor for childhood asthma, but whether susceptibility varies among school-aged children remains unclear. We aimed to investigate whether age and sex modify the association between SHS exposure and asthma in a nationally representative sample of U.S. children.

**Methods:**

We analyzed data from 5,662 children aged 6–11 years from the National Health and Nutrition Examination Survey (NHANES) 2005–2020. Asthma status was determined by self-reported physician diagnosis. SHS exposure was defined based on serum cotinine levels (≥ 0.05 ng/mL). Multivariable logistic regression was used to assess the association, adjusting for demographic, socioeconomic, and environmental factors, including age, sex, BMI, race, poverty-income ratio(PIR), household smoking, maternal smoking during pregnancy, and family history of asthma.

Multiplicative and additive interactions were examined for age and sex.

**Results:**

After adjustment for demographic, socioeconomic, and environmental factors (Model 2), SHS exposure was significantly associated with increased odds of self-reported asthma (OR = 1.28; 95% CI: 1.1–1.5). This association was significantly modified by both age (P for interaction = 0.03) and sex (*P* for interaction = 0.02). The effect was most pronounced in younger children (< 9 years; (OR = 1.59, 95% CI: 1.23–2.05) and in females (OR = 1.54, 95% CI:1.18–2.00). In contrast, the association was attenuated and non-significant in older children (≥ 9 years; OR = 1.02, 95% CI: 0.80–1.31) and in males ((OR = 1.09, 95% CI: 0.86–1.28, *P* = 0.46).

**Conclusions:**

In this cross-sectional study of school-aged children, SHS exposure was associated with higher odds of self-reported asthma. This association was modified by age and sex on the multiplicative scale, with stronger relative associations observed in younger children (< 9 years) and females. These findings underscore the need for targeted tobacco control interventions and suggest that future research should consider demographic factors as effect modifiers rather than mere confounders.

**Supplementary Information:**

The online version contains supplementary material available at https://doi.org/10.1186/s12889-026-28663-9.

## Introduction

Secondhand smoke (SHS) exposure remains a critical global public health threat. Globally, an estimated 37% of the population is exposed to SHS from burning tobacco products or exhaled smoke, with rates disproportionately higher among women and children [1, 2]. Exposure to secondhand smoke from burning tobacco products can cause sudden infant death syndrome, respiratory infections, ear infections, and asthma attacks in children [3] .From 1988 to 2014, U.S. secondhand smoke exposure among non-smokers plummeted from 87.5% to 25.2%, but then stagnated, leaving 58 million (one in four) still exposed in 2013–2014 [4]. These findings underscore that SHS exposure represents not only a medical concern but also an issue inextricably linked to health equity.

Childhood represents a critical window for pulmonary development, during which SHS exposure can inflict persistent and irreversible damage to the respiratory system [5, 6]. The World Health Organization’s recent Tobacco Knowledge Summary explicitly states that childhood SHS exposure significantly increases the risk of developing asthma, while prenatal tobacco exposure can impair fetal lung development, resulting in reduced lung function levels persisting after birth [5]. Moreover, recent research [6] demonstrates that children with daily SHS exposure exhibit not only significantly lower pulmonary function parameters—including forced vital capacity and forced expiratory volume in one second—but also higher body mass index and poorer physical fitness levels compared to unexposed children, suggesting that the health impacts of SHS exposure are multisystemic and multidimensional [7].The school-aged period (6–11 years) warrants specific attention for three reasons. First, NHANES historically designates 6–11 years as a distinct demographic group, with consistent data collection protocols across cycles [8]. Second, the 6–11 year window captures the critical transition from active alveolar development to completion, when environmental insults may exert lasting effects on lung structure and function [9, 10]. Third, children at this age increasingly spend time outside the home—in schools, playgrounds, and peers’ residences—where SHS exposure may occur beyond parental control, and lung function trajectories are established during these years [11]. Importantly, the association between SHS exposure and asthma may not be uniform across all school-aged children. Sex has been identified as a potential modifier: a study from seven northeastern cities in China found that male children with both prenatal and postnatal SHS exposure exhibited greater lung function impairment than females [12]. Age represents another potential modifier, as younger children have higher ventilation rates per unit body weight and immature metabolic enzyme systems, theoretically making them more sensitive to tobacco smoke constituents. Nevertheless, this age group has been understudied relative to infants and adolescents, leaving a critical evidence gap regarding whether age and sex modify the SHS-asthma association specifically among school-aged children (6–11 years) in a nationally representative U.S. sample.

To address this gap, this study leverages National Health and Nutrition Examination Survey (NHANES) data from 2005 to 2020 to assess whether age and sex modify the association between SHS exposure—objectively measured by serum cotinine—and asthma in children aged 6–11 years. Our findings aim to provide evidence for targeted, subgroup-specific prevention strategies.

## Methods

### Data source

One of the National Center for Health Statistics’ (NCHS) main research initiatives is NHANES. It uses a multistage, stratified probability sampling technique to evaluate.the nutritional and overall health of a non-institutionalized U.S. population using laboratory testing, physical examinations, and interviews. The NCHS Research Ethics Review Board authorized the study protocol, and all participants gave written informed consent in accordance with the STROBE criteria24 [13]. Visit https://www.cdc.gov/nchs/nhanes for detailed information on NHANES statistics.

### Study population

**Fig. 1:**
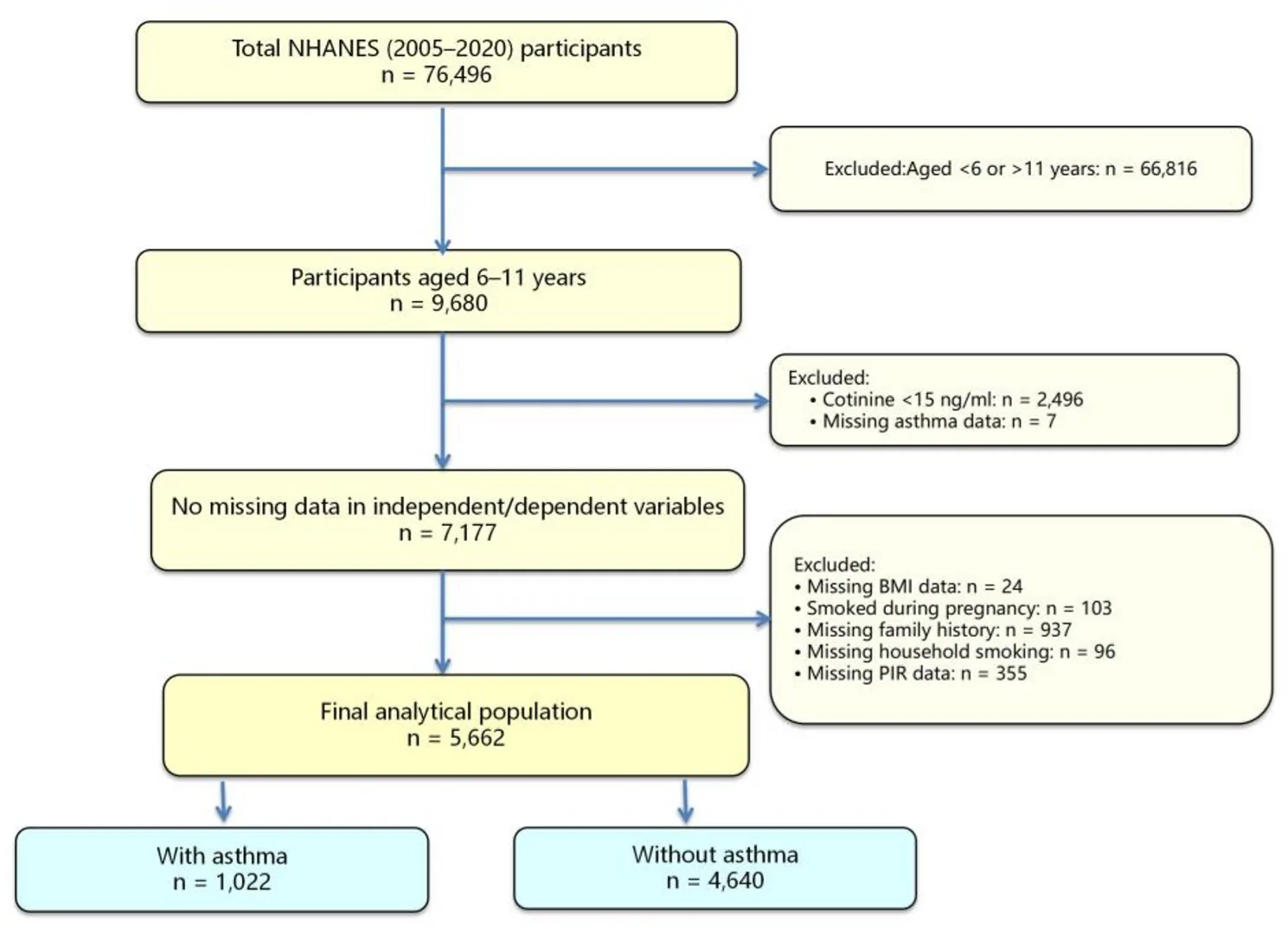
The flow diagram of the study participants, from NHANES 2005–2020

Data from the 2005–2020 cycles of the NHANES were used for this analysis.Among the 76,496 total participants, we first excluded those aged < 6 or > 11 years (*n* = 64,816), leaving 9,680 children aged 6–11 years. We then excluded participants with serum cotinine levels > 15 ng/mL (*n* = 2,496) and those with missing asthma questionnaire data (*n* = 7), resulting in 7,177 individuals with complete data on the main independent and dependent variables. Further exclusions were applied for missing data on body mass index (BMI; *n* = 24),smoking during pregnancy (*n* = 103), asthma family history (*n* = 937), household smoking (*n* = 96), and poverty-to-income ratio (PIR; *n* = 335). The final analytic sample comprised 5,662participants, including 1,020 with asthma and 4,625 without asthma (Fig. 1). Primary analyses were performed on the complete-case sample (*n* = 5,662) without survey weighting to maximize statistical power for detecting interaction effects. All subsequent analyses, including both continuous and dichotomized cotinine models, were restricted to this sample of non-active smokers (serum cotinine < 15 ng/mL).

### Assessment of tobacco smoke exposure

Serum cotinine data were obtained from the NHANES laboratory database.Cotinine, a nicotine metabolite, can signify if an individual is actively smoking or has been exposed to environmental tobacco smoke [14]. Serum cotinine levels were measured using isotope-dilution high-performance liquid chromatography combined with atmospheric pressure chemical ionization tandem mass spectrometry (ID HPLC-APCI MS/MS) [15]. To exclude the interference of active smoking in the assessment and ensure that the study subjects are appropriate for evaluating passive exposure, we defined a serum cotinine level of < 15 ng/mL as the threshold for non-active smokers based on general epidemiological criteria, as cotinine levels above 15 ng/mL are rarely achieved through secondhand smoke alone and are indicative of active smoking [16].

Given that the study participants are school-aged children aged 6–11 years, this criterion helps to identify nicotine intake resulting from household or environmental secondhand smoke exposure rather than active smoking behavior.If a kid had a detectable serum cotinine level of ≥ 0.05 ng/mL, they were deemed to have cotinine-measured exposure [17].

### Self-reported asthma

Asthma participants were identified through their responses to the Medical Condition Questionnaire, specifically the item: “Has a doctor or other health professional ever told you that you have asthma? [18] ”Thus, asthma status in this study reflects self-reported physician diagnosis rather than independently verified cases.

### Covariables

Covariates included in the analysis were selected based on prior literature and clinical relevance to account for potential confounding in the association between secondhand smoke exposure and childhood asthma [19, 20]. Demographic covariates included age, sex, race (categorized as Non-Hispanic White, Non-Hispanic Black, Mexican American, and Other), and poverty-to-income ratio (PIR), which was classified into three categories (< 1.30, 1.31–3.50, and > 3.50) representing low, middle, and high family income levels. Body mass index (BMI) was calculated as weight in kilograms divided by height in meters squared and modeled as a continuous variable.Environmental and familial factors comprised household smoking exposure, maternal smoking during pregnancy, and family history of asthma, all of which were obtained from questionnaire data. Hematological parameters (white blood cell count, eosinophil percentage, platelet count, hemoglobin, and systemic immune-inflammation index) were presented in the baseline characteristics (Table 1) for descriptive purposes but were not included as covariates in the primary regression models. This decision was made because these markers may lie on the causal pathway between SHS exposure and asthma, and adjusting for potential mediators could introduce overadjustment bias.

**Table 1:** Baseline characteristics of children stratified by cotinine levels

Variables	Total (*N* = 5662)	Cotinine< 0.05 ng/mL (*N*= 3224)	Cotinine> 0.05 ng/mL (*N* = 2438)	*p*
Demographic characteristics				
Age, years	8.6 ± 1.7	8.6 ± 1.7	8.5 ± 1.7	0.008
BMI, kg/m^2^	19.0 ± 4.5	18.9 ± 4.3	19.2 ± 4.7	0.018
Sex, *n* (%)				0.796
Male	2877 (50.8)	1643 (51)	1234 (50.6)	
Female	2785 (49.2)	1581 (49)	1204 (49.4)	
Race, *n* (%)				< 0.001
Non-Hispanic White	1613 (28.5)	838 (26)	775 (31.8)	
Non-Hispanic Black	1394 (24.6)	487 (15.1)	907 (37.2)	
Mexican American	1337 (23.6)	1028 (31.9)	309 (12.7)	
Other	1318 (23.3)	871 (27)	447 (18.3)	
PIR, *n* (%)				< 0.001
<1.30	1993 (35.2)	837 (26)	1156 (47.4)	
1.31-3.50	2598 (45.9)	1495 (46.4)	1103 (45.2)	
>3.50	1071 (18.9)	892 (27.7)	179 (7.3)	
SHS exposure characteristics				
Household Smoking, *n *(%)				< 0.001
No	4410 (77.9)	3007 (93.3)	1403 (57.5)	
Yes	1252 (22.1)	217 (6.7)	1035 (42.5)	
Maternal smoking during pregnancy, *n* (%)				< 0.001
No	4973 (87.8)	3122 (96.8)	1851 (75.9)	
Yes	689 (12.2)	102 (3.2)	587 (24.1)	
Asthma-related characteristics				
Family history of asthma, *n* (%)				< 0.001
No	3807 (67.2)	2307 (71.6)	1500 (61.5)	
Yes	1855 (32.8)	917 (28.4)	938 (38.5)	
Self-reported asthma, *n* (%)				< 0.001
No	4640 (81.9)	2725 (84.5)	1915 (78.5)	
Yes	1022 (18.1)	499 (15.5)	523 (21.5)	
Hematological parameters				
WBC, 109 /l	7.1 ± 2.1	7.2 ± 2.0	7.0 ± 2.1	< 0.001
EOS, %	2.8 (1.7, 5.1)	2.7 (1.7, 4.9)	2.9 (1.8, 5.2)	0.002
PLT,109 /l	293.4 ± 63.8	290.4 ± 63.0	297.2 ± 64.7	< 0.001
HGB,109 /l	13.0 ± 0.9	13.1 ± 0.8	12.9 ± 0.9	< 0.001
SII^a^	5.9 ± 0.5	5.9 ± 0.5	5.9 ± 0.5	0.444

*Abbreviations*: *PIR *poverty-to-income ratio, *BMI *body mass index, *WBC *white blood cell count, *EOS *eosinophil percentage, *PLT *platelet count, *HGB *hemoglobin, *lnSII *systemic immune-inflammation index (natural log-transformed)

SII^a^ represents the natural log-transformed systemic immune-inflammation index

### Statistical analysis

For continuous variables, the mean (standard deviation, SD) and median (interquartile range, IQR) were computed. All categorical variables were described in frequencies (n) and percentage (%). The analysis of variance (ANOVA) and the Chi-square test were conducted to evaluate the trends in continuous and categorical variables, respectively.

Missing covariate data were handled using multiple imputation by chained equations (MICE) with 5 imputations and 20 iterations. Primary analyses were conducted without survey weighting to maximize statistical power for subgroup and interaction analyses, which are inherently sample-demanding.Multivariable logistic regression models were employed to evaluate the association between serum cotinine levels and childhood asthma. Logistic regression is a standard approach for binary outcome data in epidemiological research, providing interpretable odds ratios that approximate relative risks when the outcome is rare [21, 22]. Cotinine was analyzed both as a continuous variable (natural log-transformed to improve model fit) and as a dichotomous variable (exposure group: cotinine ≥ 0.05 ng/mL vs. non-exposure group: cotinine < 0.05 ng/mL).This cut-off corresponds to the limit of detection (LOD) for serum cotinine in early NHANES cycles and has been widely used in epidemiologic studies to define detectable secondhand smoke exposure [23]. Children with cotinine levels below this threshold are considered to have no measurable exposure. Two sequential adjustment models were constructed: Model 1 adjusted for demographic factors including age, sex, race, BMI, and PIR; Model 2 further adjusted for environmental and familial factors including household smoking exposure, maternal smoking during pregnancy, and family history of asthma.Multicollinearity among independent variables was assessed using variance inflation factors (VIF), with a threshold of < 2.0 indicating no significant collinearity [24]. Subgroup analyses were stratified by age, sex, BMI category, family history of asthma and SII level. Because alveolar maturation is largely completed by approximately 9 years of age [25], and to ensure balanced sample sizes for subgroup analyses (children < 9 years, *n* = 2,728; ≥9 years, *n* = 2,917), we dichotomized age at 9 years.To flexibly model the dose-response relationship between log-transformed cotinine and asthma, we applied restricted cubic splines with three knots placed at the 10th, 50th, and 90th percentiles in logistic regression models, stratified by age group and sex. The reference value for cotinine was set at 0.05 ng/mL (the exposure threshold).Multiplicative interaction was tested by including product terms in models, with significance determined by likelihood ratio tests. Additive interaction was evaluated using RERI, AP, and SI with 95% CIs calculated via the delta method [26]. We conducted several sensitivity analyses to assess the robustness of our primary findings. First, to evaluate whether adjustment for hematological parameters would materially alter the observed associations, we re-ran the primary models with additional adjustment for white blood cell count, eosinophil percentage, platelet count, hemoglobin, and systemic immune-inflammation index using the complete-case sample (*n* = 5,662). Second, to address potential selection bias from excluding participants with missing covariate data, we performed multiple imputation by chained equations (MICE, 5 imputations, 20 iterations) and re-ran the primary models on the imputed dataset (*n* = 7,177). Third, to account for the complex survey design of NHANES, we incorporated sampling weights, strata, and primary sampling units (PSUs) into the multiply imputed models(*n* = 7,177), following NCHS analytic guidelines with 2-year examination weights divided by the number of cycles (weight/8). Fourth, to examine whether the choice of the 9-year age cutoff influenced our findings, we performed sensitivity analyses using alternative cutoffs at 8 and 10 years.

Statistical analyses were conducted using R Statistical Software (Version 4.2.2, http://www.R-project.org, The R Foundation) and the Free Statistics analysis platform (Version 1.9, Beijing, China, http://www.clinicalscientists.cn/freestatistics). A two-sided *p*-value below 0.05 was deemed statistically significant.

## Results

### Baseline characteristics by cotinine levels

A total of 5,662 children aged 6–11 years from the NHANES 2005–2020 cycles were included in the final analysis, with 1,022 (18.0%) having physician-diagnosed asthma, comprising 3,224 in the non-exposure group (cotinine < 0.05 ng/mL) and 2,438 in the exposure group(cotinine ≥ 0.05 ng/mL). The demographic and clinical characteristics of participants stratified by cotinine levels are summarized in Table 1. Compared to the non-exposure group, children in the exposure group had slightly higher BMI (19.2 ± 4.7 vs. 18.9 ± 4.3, *p* = 0.018), were more likely to be from low-income families (PIR < 1.30: 47.4% vs. 26.0%, *p* < 0.001), and had higher prevalence of family asthma history (38.5% vs. 28.4%, *p* < 0.001) and household smoking exposure (42.5% vs. 6.7%, *p* < 0.001). Significant differences were also observed in racial distribution and multiple hematological parameters ( *p* < 0.05), except for lnSII (*p* = 0.444).

### Association between household smoke exposure and childhood asthma

There was no alarming collinearity between the independent variables in the logistic regression models, according to multicollinearity diagnostics.In the crude model, cotinine (as a continuous variable) was positively associated with asthma (OR = 1.31, 95% CI: 1.21–1.43, *P* < 0.001). This association remained significant after sequential adjustment for demographic and environmental in Models 1 to 2 (Model 2: OR = 1.22, 95% CI: 1.08–1.38, *P* = 0.002).When cotinine was analyzed as a dichotomized variable, children in the exposure group (cotinine ≥ 0.05 ng/mL) had significantly higher odds of asthma compared to those in the non-exposure group (cotinine < 0.05 ng/mL) across all models (Model 2: OR = 1.27, 95% CI: 1.06–1.51, *P* = 0.008) (Table 2).

**Table 2:** Association between cotinine levels and childhood asthma risk

Variable	Non-adjusted		Model 1		Model 2	
	OR (95% CIs)	*p*	OR (95% CIs)	*p*	OR (95% CIs)	*p*
cotinine	1.31 (1.21 ~ 1.43)	< 0.001	1.27 (1.15 ~ 1.4)	< 0.001	1.22 (1.08 ~ 1.38)	0.002
cotinine < 0.05ng/mL	1(Ref)		1(Ref)		1(Ref)	
cotinine ≥ 0.05ng/mL	1.49 (1.3 ~ 1.71)	< 0.001	1.39 (1.19 ~ 1.62)	< 0.001	1.27 (1.06 ~ 1.51)	0.008

values are presented as odds ratio (95% confidence interval)

Model 1: adjusted age, gender,race,BMI,PIR

Model 2: adjusted Model 1+ Maternal smoking during pregnancy,Household Smoking,Family history of asthma

### Subgroup analyses

**Fig. 2:**
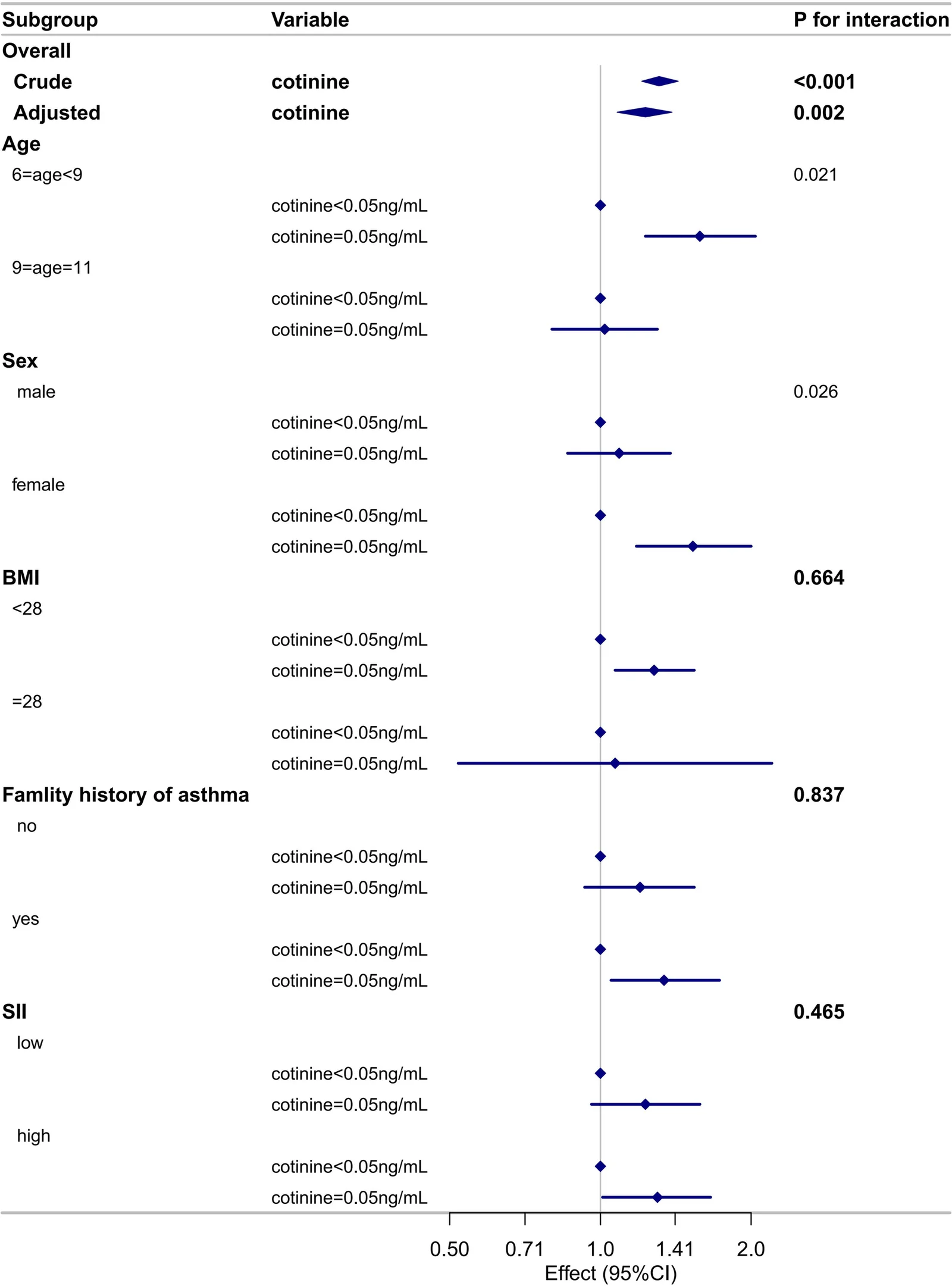
Forest plot of subgroup analyses for the association between cotinine exposure and childhood asthma. Data are presented as odds ratios (ORs) and 95% confidence intervals (CIs). SII^a^ represents the natural log-transformed systemic immune-inflammation index. For subgroup, SII^a^ values were dichotomized at the median into low (below median) and high (above median) group

In subgroup analyses stratified by age, sex, BMI, family history of asthma, and SII level, significant interaction effects were observed between household smoke exposure and both age (*P* = 0.021) and sex (*P* = 0.025),indicating that the association between secondhand smoke and Childhood asthma is modified by these demographic factors (Fig. 2). When stratified by age group, the association between cotinine exposure and asthma risk was more pronounced in younger children. Among children aged < 9 years, those in the exposure group (cotinine ≥ 0.05 ng/mL) had significantly higher odds of asthma compared to the non-exposure group (OR = 1.59, 95% CI: 1.23–2.05, *p* < 0.001). In contrast, among children aged ≥ 9 years, the association was attenuated and did not reach statistical significance (OR = 1.02, 95% CI: 0.80–1.31, *p* = 0.752). Among female children, cotinine exposure was significantly associated with increased odds of asthma (OR = 1.54, 95% CI:1.18–2.00, *p* < 0.001). Among male children, the association was attenuated and did not reach statistical significance (OR = 1.09 95% CI: 0.86–1.28, *p* = 0.46).

### Stratified analyses by age and sex

**Fig. 3:**
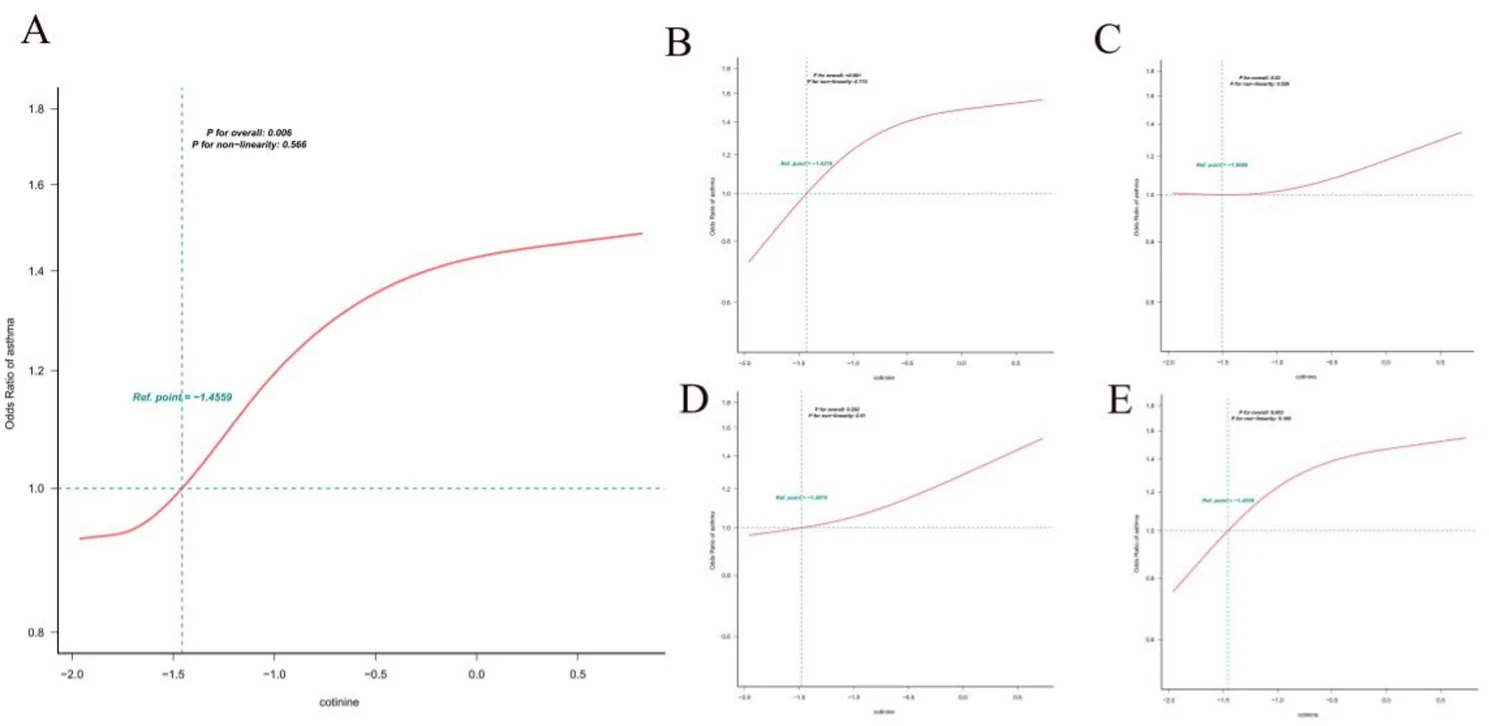
Logistic regression curves for the association between cotinine levels and asthma risk. **A** ALL Children; (**B**) Children aged < 9 years; (**C**) Children aged ≥ 9 years; (**D**) Male children; (**E**) Female children

Linear relationships were observed in the fitted logistic regression curves for the overall population, with distinct patterns between younger and older children, as well as between males and females. In children aged < 9 years, the exposure-response curve suggested a positive association between cotinine levels and asthma risk, whereas in those aged ≥ 9 years, the association appeared attenuated (see fitted curves for age < 9 and age ≥ 9). Similarly, sex-stratified analyses revealed that the association was more pronounced in females compared to males, with females showing a steeper increase in asthma risk with increasing cotinine levels (Fig. 3).

### Interaction analysis

**Fig. 4:**
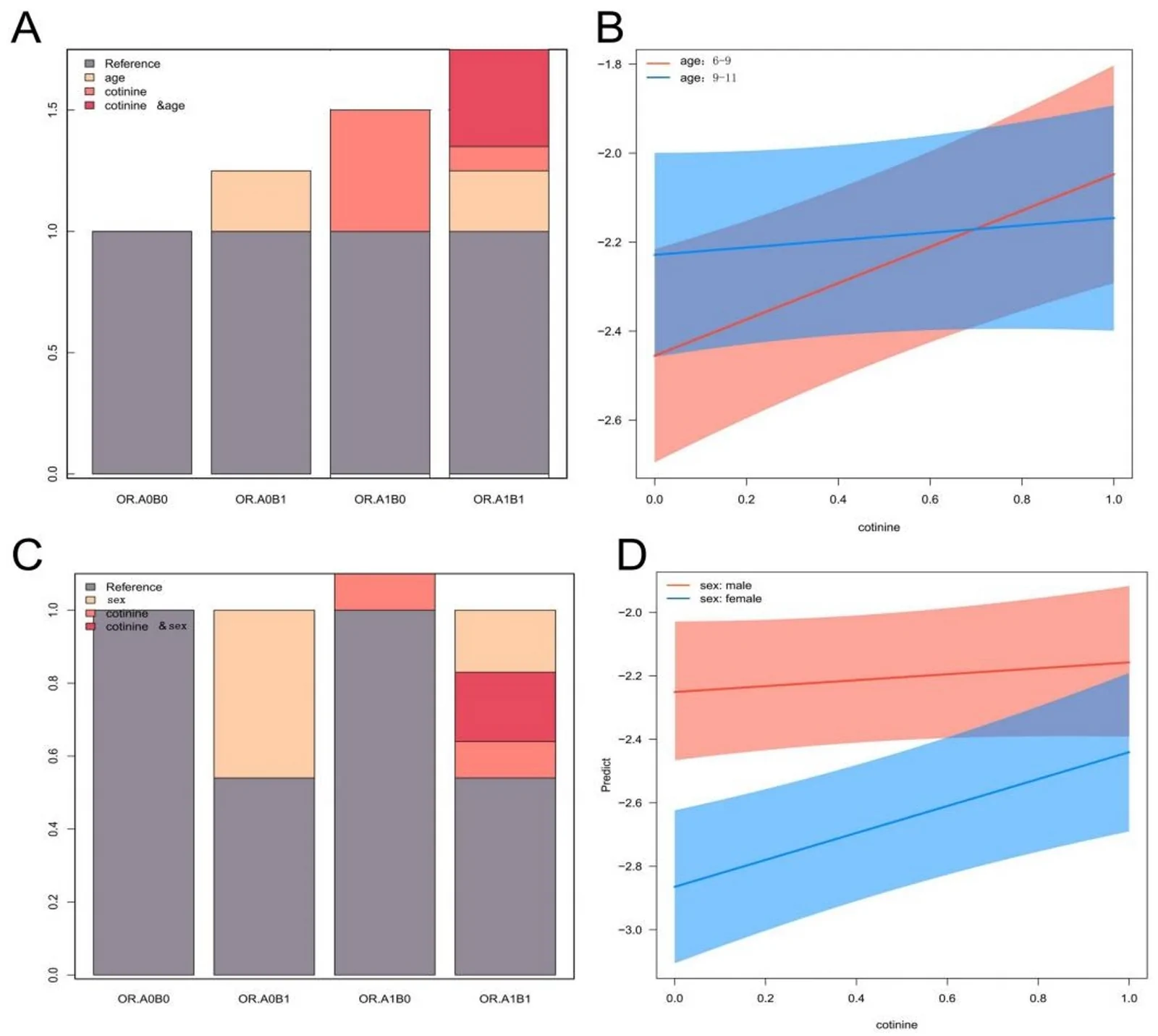
Subgroup-specific associations between secondhand smoke exposure and self-reported asthma stratified by age and sex. This figure visualizes the interaction effects of secondhand smoke (SHS) exposure, age, and sex on childhood asthma, complementing the data presented in Table 3. **A** shows odds ratios (ORs) for SHS exposure across age subgroups; **B** shows logistic regression curves for the association between SHS exposure and asthma by age group; **C** shows ORs for SHS exposure by sex; **D** presents logistic regression curves by sex

The interaction analyses further quantified the effect modification observed in subgroup analyses. On the multiplicative scale, the interaction term for age was 0.72 (95% CI: 0.54–0.97,*P* = 0.03), and for sex was 1.40 (95% CI: 1.04–1.87,*P* = 0.02), indicating statistically significant effect modification by both factors. Specifically, the association between SHS exposure and asthma was stronger in children aged < 9 years than in those aged ≥ 9 years, and stronger in females than in males.Additive interaction measures were largely non-significant, suggesting that the modification operates mainly on the relative risk scale (Table 3; Fig. 4). These findings suggest that age and sex may modify the association between secondhand smoke exposure and Childhood asthma with younger children and females being more vulnerable to the adverse effects of cotinine.

**Table 3:** Multiplicative and additive interaction of age and sex on the association between secondhand smoke exposure and childhood asthma

	Age	Sex
Multiplicative interaction		
Multiplicative scale	0.72(0.54 ~ 0.97)	1.4(1.04 ~ 1.87)
*p*	0.03	0.02
Additive interaction		
RERI (95% CI)	−0.39(−0.78~−0.01)	0.21(−0.04 ~ 0.45)
AP (95% CI)	−0.31(−0.64 ~ 0.01)	0.24(−0.05 ~ 0.52)
SI (95% CI)	0.4(0.13–1.27)	NA

*Abbreviations*: *OR* Odds ratio, *CI* Confidence interval, *RERI* Relative excess risk due to interaction, *AP* Attributable proportion, *SI* Synergy index, *NA* Not applicable

### Sensitivity analyses

The robustness of our primary findings was supported by several sensitivity. First, to evaluate whether adjustment for hematological parameters would materially alter the observed associations, we re-ran the primary models with additional adjustment for white blood cell count, eosinophil percentage, platelet count, hemoglobin, and systemic immune-inflammation index using the complete-case sample (*n* = 5,662). The effect estimates remained virtually unchanged from Model 2 to the hematological-adjusted model (Supplementary Table S1), supporting that these markers do not substantially confound the observed association and are not needed in the primary models. Second, to address potential selection bias from excluding participants with missing covariate data, we performed multiple imputation by chained equations and re-ran the primary models on the imputed dataset (*n* = 7,177). The association remained significant (OR = 1.28, 95% CI: 1.10–1.49), and the multiplicative interaction remained significant for sex (*P* = 0.01) and was borderline significant for age (*P* = 0.068), with point estimates and direction of associations consistent with the primary analysis (Supplementary Tables S2–S3).Third, when incorporating NHANES survey weights into the complete-case models (*n* = 5,662), the main association remained positive (OR = 1.22, 95% CI: 1.00–1.48) (Supplementary Table S4).Fourth, to examine whether the choice of the 9-year age cutoff influenced our findings, we performed sensitivity analyses using alternative cutoffs at 8 and 10 years. The pattern of effect modification by age was confirmed across all specifications, with multiplicative interactions remaining significant (P for interaction: 0.005 for 8-year cutoff; 0.044 for 10-year cutoff) (Supplementary Table S5). Collectively, these sensitivity analyses supported the robustness of our primary findings, with consistent direction and magnitude of effect estimates across all analytic approaches.

## Discussion

This population-based study of 5,662 school-aged children aged 6–11 years from the NHANES 2005–2020 cycles provides compelling evidence that secondhand smoke exposure, objectively measured by serum cotinine, is significantly associated with increased odds of self-reported asthma. Given the cross-sectional design of NHANES, our findings represent an association between current SHS exposure (measured by serum cotinine) and prevalent self-reported asthma, rather than a causal effect. While reverse causality is unlikely given that asthma diagnosis typically precedes cotinine measurement, we cannot establish the temporal sequence with certainty. Notably, our findings extend beyond confirming this association by demonstrating that even within this narrow school-aged range, age and sex serve as critical effect modifiers: the adverse effects of SHS exposure were more pronounced in younger school-aged children (< 9 years) and in females. These results underscore the importance of recognizing differential vulnerability within the school-aged pediatric population and moving toward more nuanced, subgroup-specific approaches in both research and public health practice.

The overall positive association between SHS exposure and childhood asthma observed in our study aligns with a substantial body of evidence. A comprehensive meta-analysis by He et al., synthesizing 93 studies (including 41 cohort studies) from 1975 to 2020, demonstrated a significant positive association between secondhand smoke exposure and physician-diagnosed childhood asthma, with a pooled odds ratio of 1.24 (95% CI: 1.20–1.28) [27]. Similarly, the World Health Organization’s Tobacco Knowledge Summary explicitly identifies childhood SHS exposure as a causal risk factor for asthma onset [4]. Our findings, derived from a large, nationally representative sample of school-aged children with objective biomarker-based exposure assessment, further strengthen this evidence base. The persistence of the association after comprehensive adjustment for demographic, environmental, family history (Model 2: OR = 1.22, 95% CI: 1.08–1.38 for continuous cotinine) suggests an independent effect of SHS exposure on asthma, not readily explained by confounding factors.

The most novel contribution of our study lies in the identification of age and sex as modifiers of this association within the school-aged population. The significantly stronger effect observed in children aged < 9 years compared to those aged ≥ 9 years (interaction *P* = 0.03) has important implications for understanding vulnerability during the school-age period.Younger school-aged children have higher ventilation rates per unit body weight, leading to greater inhaled doses of tobacco smoke constituents relative to older children [28], as children’s developing physiology—including higher respiratory rates and immature metabolic pathways—renders them particularly susceptible to environmental pollutants. Additionally, their immune and respiratory systems are still undergoing active maturation during early school years, with developing antioxidant defense mechanisms and metabolizing enzyme systems that may be less capable of detoxifying harmful smoke components [29]. Our finding of greater asthma susceptibility in females (interaction *p* = 0.02) differs from the Seven Northeastern Cities study in China, which reported greater lung function impairment in males [7]. This discrepancy may be explained by differences in outcome measures (asthma diagnosis vs. lung function parameters), population characteristics (U.S. vs. China), or exposure assessment methods (serum cotinine vs. questionnaire). Interestingly, a birth cohort study using hair cotinine also found that girls with early-life allergic sensitization had greater SHS-related lung function deficits than boys [23], which aligns with our results. These contrasting findings suggest that sex-based susceptibility to SHS may be context-dependent, varying by co-exposures (e.g., allergens), outcome measures, and potentially by genetic background. Future research should examine whether the modifying effect of sex differs between atopic and non-atopic children. Several biological mechanisms may explain the greater susceptibility to SHS-associated asthma observed in school-aged females, although these mechanisms were not directly tested in our study and remain hypotheses for future investigation. Sex hormones modulate immune responses in a sex-dependent manner. Estrogen enhances type 2 inflammation, increases eosinophilic infiltration, and upregulates IL-4 and IL-13 expression, leading to greater airway hyperreactivity. Conversely, testosterone exerts protective effects by dampening Th2-driven inflammation [30]. In addition, androgens negatively regulate type 2 immune responses sustained by CD4 + Th2 cells and group 2 innate lymphoid cells (ILC2s), diminishing allergic airway inflammation and providing relative protection in males [31]. We emphasize that these mechanistic explanations are speculative in the context of our study, as we did not directly measure hormonal or immune parameters. Future research integrating biomarker data with clinical outcomes is needed to confirm these pathways and elucidate the biological basis of the observed susceptibility differences.Interestingly, while we observed significant multiplicative interactions for both age and sex, the additive interaction measures did not reach statistical significance. This discrepancy suggests that the effect modification operates primarily on a relative, rather than an absolute, risk scale. From a public health perspective, the absence of additive interaction implies that the absolute risk difference attributable to SHS exposure does not differ significantly across age or sex strata, even though the relative odds differ. However, given the limited statistical power for additive interaction testing, the lack of significance should be interpreted cautiously; larger studies are needed to confirm these findings. This nuance is important for interpreting the clinical and population-level impact of our findings, as absolute measures are often more relevant for risk communication and resource allocation. Future studies with larger sample sizes or longitudinal designs are warranted to further explore these complex effect modification patterns.

Sensitivity analyses supported the robustness of our findings. Additional adjustment for hematological parameters did not materially change the effect estimates, suggesting that these markers are not major confounders and need not be included in the primary models. Multiple imputation for missing covariates yielded point estimates and direction of associations consistent with the complete-case analysis, indicating that excluding participants with missing data did not substantially bias our main conclusions. Weighted analyses incorporating NHANES survey weights further confirmed the positive main association. In addition, sensitivity analyses using alternative cutoffs at 8 and 10 years consistently supported the modifying role of age, with the 9-year cutoff providing the most balanced subgroup sample sizes and thus serving as the most reasonable age stratification for the primary analysis.

This study has several notable strengths. First, the use of serum cotinine as an objective biomarker of SHS exposure eliminates recall bias inherent in self-reported exposure assessment and provides a quantitative measure of internal dose specifically in school-aged children. Second, the large, nationally representative sample of U.S. school-aged children from multiple NHANES cycles enhances generalizability and statistical power for subgroup analyses within this age range. Third, the comprehensive adjustment for a wide range of potential confounders—including demographic, environmental, and familial—reduces the likelihood of residual confounding. Fourth, the systematic evaluation of both multiplicative and additive interactions provides a rigorous assessment of effect modification specifically within the school-aged population.

Several limitations should also be acknowledged. First, the cross-sectional design precludes causal inference; while we observed associations between current cotinine levels and ever-diagnosed asthma in school-aged children, we cannot establish temporal relationships or rule out reverse causality. The findings should therefore be interpreted as an association between current SHS exposure and prevalent self-reported asthma. Future longitudinal studies with repeated exposure measurements are needed to clarify the temporal relationship and determine whether SHS exposure precedes asthma onset or exacerbates existing disease. Second, asthma status was based on self-reported physician diagnosis rather than objective diagnostic criteria such as spirometry or bronchial challenge testing, which may introduce misclassification bias. Future studies incorporating objective pulmonary function testing and phenotyping are warranted. Third, serum cotinine reflects relatively recent exposure (approximately 2–3 days) and may not capture cumulative lifetime exposure or exposure patterns throughout the school-age years, potentially underestimating long-term effects. Fourth, despite extensive covariate adjustment, unmeasured confounding—such as detailed dietary factors, indoor air pollution beyond tobacco smoke, or genetic polymorphisms affecting smoke metabolism—cannot be entirely excluded.

Our findings have several important implications specifically for the health and well-being of school-aged children. First, from a clinical perspective, pediatricians and school health professionals should recognize that not all school-aged children are equally susceptible to SHS exposure; younger school-aged children (6–9 years) and females may require enhanced monitoring and more aggressive counseling regarding smoke exposure reduction. From a policy perspective, the benefits of smoke-free legislation for respiratory health extend not only to adults [32] but also to children.Thus, the combination of clinical monitoring and policy interventions represents an effective strategy to protect high-risk school-aged children from the harms of SHS exposure. Second, routine inquiry about household smoking status during school health screenings and, where feasible, objective exposure assessment (e.g., salivary or urinary cotinine) could help identify high-risk school-aged children for targeted intervention. School nurses are uniquely positioned to identify children with asthma who may be experiencing SHS exposure and to provide education to families. Third, asthma control remains far from optimal worldwide.Racine et al. identified current smoking and poor lung function as significant predictors of uncontrolled asthma in a real-world cohort study [33]. These findings further underscore the importance of identifying modifiable risk factors for improving asthma control, particularly tobacco exposure. Our results complement this evidence by identifying younger school-aged children and females as particularly susceptible subgroups, suggesting that tobacco control interventions should be optimized for these high-risk populations.

From a research perspective, our findings highlight the importance of conducting studies specifically focused on the school-aged developmental period rather than combining broad pediatric age ranges. Future studies should incorporate longitudinal designs following children throughout the school years to establish temporality, utilize repeated exposure measurements to capture cumulative effects during this critical period, and explore underlying mechanisms through integration of biomarker data with clinical outcomes. Gene-environment interaction studies focusing specifically on school-aged children may further elucidate the biological basis of observed susceptibility differences.

## Conclusions

Secondhand smoke exposure is associated with childhood asthma, but this association is significantly modified by age and sex. Younger school-aged children and females are particularly susceptible, highlighting the importance of subgroup-specific prevention strategies in clinical and public health practice.

## Supplementary Information

Supplementary Material 1.

## Data Availability

The datasets used in this study are publicly available from the National Health and Nutrition Examination Survey (NHANES) database.The data can be accessed at: https:// www.cdc.gov/nchs/nhanes/.

## Abbreviations

BMI: Body mass index
CI: Confidence interval
EOS: Eosinophil percentage
HGB: Hemoglobin
MICE: Multiple imputation by chained equations
NCHS: National Center for Health Statistics
NHANES: National Health and Nutrition Examination Survey
PIR: Poverty-to-income ratio
PLT: Platelet count
PSU: Primary sampling unit
RERI: Relative excess risk due to interaction
SHS: Secondhand smoke
SII: Systemic immune-inflammation index
VIF: Variance inflation factor
WBC: White blood cell count
