# Supplementary material for: Age and sex modify the association between secondhand smoke exposure and self-reported asthma in school-aged children (6–11 years): a cross-sectional study from NHANES 2005–2020
Source: BMC Public Health. 2026 Aug 3;26:3118. doi: 10.1186/s12889-026-28663-9 (PMC13721588; doi:10.1186/s12889-026-28663-9)
Supplement: Supplementary file 1 [file 12889_2026_28663_MOESM1_ESM.docx]

# **SUPPLEMENTARY MATERIALS**

# Supplementary Tables for: Age and Sex Modify the Association Between Secondhand Smoke Exposure and Self-Reported Asthma in School-Aged Children (6–11 Years)

### Supplementary Table S1. Sensitivity Analysis with Additional Adjustment for Hematological Parameters (n = 5,662)

| **Variable** | **Model 2** | | **Model 2 + Hematological** | | |
| --- | --- | --- | --- | --- | --- |
|  | **OR (95% CIs)** | **p** | | **OR (95% CIs)** | **p** |
| cotinine | 1.22 (1.08~1.38) | 0.002 | 1.23 (1.08~1.39) | | 0.002 |
| cotinine<0.05ng/mL | 1(Ref) |  | 1(Ref) | |  |
| cotinine≥0.05ng/mL | 1.27 (1.06~1.51) | 0.008 | 1.27 (1.06~1.51) | | 0.008 |

 Results are based on the complete-case sample (n = 5,662). Model 2 adjusted for age, sex, race, BMI, PIR, maternal smoking during pregnancy, household smoking, and family history of asthma. Model 2 + Hematological adjusted for all variables in Model 2 plus white blood cell count, eosinophil percentage, platelet count, hemoglobin, and systemic immune-inflammation index (lnSII).

**Supplementary Table S2. Multiple Imputation Analysis: Association Between Cotinine Levels and Childhood Asthma Risk (n = 7,177)**

| **Variable** | **Non-adjusted** | | **Model 1** | | **Model 2** | |
| --- | --- | --- | --- | --- | --- | --- |
|  | **OR (95% CIs)** | **p** | **OR (95% CIs)** | **p** | **OR**  **(95% CIs)** | **p** |
| cotinine | 1.09 (1.04~1.15) | 0.001 | 1.06 (1~1.12) | 0.044 | 1.02 (0.95~1.09) | 0.611 |
| cotinine<0.05ng/mL | 1(Ref) |  | 1(Ref) |  | 1(Ref) |  |
| cotinine≥0.05ng/mL | 1.52 (1.34~1.71) | <0.001 | 1.41 (1.23~1.62) | <0.001 | 1.28 (1.1~1.5) | 0.002 |

Values are presented as odds ratio (95% confidence interval).

Model 1: adjusted for age, sex, race, BMI, and PIR.

Model 2: adjusted for Model 1 + maternal smoking during pregnancy, household smoking, and family history of asthma.

### **Supplementary Table S3.Multiple Imputation Analysis: Multiplicative and Additive Interaction of Age and Sex (n = 7,177)**

|  | Age | Sex |
| --- | --- | --- |
| Multiplicative interaction |  |  |
| Multiplicative scale | 0.78（0.60~1.02） | 1.38(1.06~1.79) |
| p | 0.06 | 0.02 |
| Additive interaction |  |  |
| RERI (95% CI) | -0.27(-0.64~-0.09) | 0.16(-0.05~0.38) |
| AP (95% CI) | -0.18(-0.44~0.08) | 0.20(-0.07~0.48) |
| SI (95% CI) | 0.65(0.35-1.19) | NA |

Abbreviations: OR, odds ratio; CI, confidence interval; RERI, relative excess risk due to interaction; AP, attributable proportion; SI, synergy index; NA, not applicable.

### **Supplementary Table S4.Weighted Analysis: Association Between Cotinine Levels and Childhood Asthma Risk (n = 7,177)**

| **Variable** | **Non-adjusted** | | **Model 1** | | **Model 2** | |
| --- | --- | --- | --- | --- | --- | --- |
|  | **OR (95% CIs)** | **p** | **OR (95% CIs)** | **p** | **OR**  **(95% CIs)** | **p** |
| cotinine | 1.12(1.04~1.20) | 0.003 | 1.14 (1.03~1.19) | 0.005 | 1.04(0.97~1.13) | 0.25 |
| cotinine<0.05ng/mL | 1(Ref) |  | 1(Ref) |  | 1(Ref) |  |
| cotinine≥0.05ng/mL | 1.38(1.18~1.61) | <0.001 | 1.35 (1.14~1.60) | <0.001 | 1.24(1.10~1.50) | 0.035 |

Results are based on the complete-case sample (n = 7,177). All estimates are weighted to account for the complex survey design of NHANES, incorporating sampling weights (WTMEC2YR divided by the number of cycles), stratification variables (SDMVSTRA), and primary sampling units (SDMVPSU).

Model 1 adjusted for age, sex, race, BMI, and PIR.

Model 2 adjusted for Model 1 + maternal smoking during pregnancy, household smoking, and family history of asthma.

### Supplementary Table S5. Subgroup Analyses for Age Cutoff Sensitivity: Age-Stratified Associations Between SHS Exposure and Asthma Under Alternative Age Specifications（n=7,177）

| Age cutoff | Subgroup | Case/Total | OR(95%CI) | P | P for interaction |
| --- | --- | --- | --- | --- | --- |
| 8 years |  |  |  |  | 0.005 |
| 6-8 years | cotinine<0.05ng/mL | 117/1007 | Reference |  |  |
|  | cotinine≥0.05ng/mL | 164/775 | 1.98(1.31, 2.99) | 0.002 |  |
| 8-11 years | cotinine<0.05ng/mL | 382/2211 | Reference |  |  |
|  | cotinine≥0.05ng/mL | 357/1652 | 1.02(0.79, 1.33) | 0.87 |  |
| 9 years |  |  |  |  | 0.002 |
| 6-9 years | cotinine<0.05ng/mL | 202/1514 | Reference |  |  |
|  | cotinine≥0.05ng/mL | 254/1214 | 1.87(1.37, 2.55) | <0.001 |  |
| 9-11 years | cotinine<0.05ng/mL | 297/1704 | Reference |  |  |
|  | cotinine≥0.05ng/mL | 267/1213 | 0.87(0.65, 1.17) | 0.36 |  |
| 10 years |  |  |  |  | 0.044 |
| 6-10 years | cotinine<0.05ng/mL | 289/2042 | Reference |  |  |
|  | cotinine≥0.05ng/mL | 338/1622 | 1.55(1.17, 2.04) | 0.002 |  |
| 10-11 years | cotinine<0.05ng/mL | 210/1176 | Reference |  |  |
|  | cotinine≥0.05ng/mL | 183/805 | 0.83(0.58, 1.20) | 0.32 |  |

Abbreviations: CI, confidence interval; OR, odds ratio; SHS, secondhand smoke.

Notes: For each cutoff (8, 9, and 10 years), the table presents the association between SHS exposure (cotinine ≥0.05 ng/mL vs. <0.05 ng/mL) and self-reported asthma within each age stratum.
